# Swin-WaveNet: frequency-guided boundary restoration for robust skin lesion segmentation

**DOI:** 10.1186/s42492-026-00235-z

**Published:** 2026-10-19

**Authors:** Zhiping Dan, Run Zhang, Tinglong Tang, Yirong Wu, Xiaosheng Yu, Shuifa Sun, Ben Wang

**Affiliations:** 1 Hubei Key Laboratory of Intelligent Vision Based Monitoring for Hydroelectric Engineering, China Three Gorges University, Yichang, Hubei 443002 China; 2 College of Computer and Information Technology, China Three Gorges University, Yichang, Hubei 443002 China; 3 Yichang Key Laboratory of Intelligent Medicine, China Three Gorges University, Yichang, Hubei 443002 China; 4 Institute of Advanced Studies in Humanities and Social Sciences, Beijing Normal University, Zhuhai, Guangdong 519087 China; 5 School of Information Science and Technology, Hangzhou Normal University, Hangzhou, Zhejiang 311121 China

**Keywords:** Skin lesion segmentation, Dermoscopic image analysis, Wavelet decomposition, State-space modeling, Boundary-aware learning

## Abstract

Accurate skin lesion segmentation remains difficult when dermoscopic boundaries are fragmented by hair occlusion, uneven illumination, or weak local contrast. To address this problem, the authors propose Swin-WaveNet, a frequency-guided encoder-decoder that combines a Swin Transformer backbone with wavelet decomposition and selective state-space modeling. Rather than passing skip features directly to the decoder, the proposed method separates low-frequency semantic information from high-frequency details, selectively refines boundary-related responses, and suppresses artifact-induced activation prior to feature reconstruction. The main results are reported as the mean and standard deviation of the sample over three runs. Swin-WaveNet achieved a Dice score of 89.69% ± 0.51% and a 95th-percentile Hausdorff distance (HD95) of *23.60 ± 1.04* pixels on the International Skin Imaging Collaboration (ISIC) 2018 dataset. Without fine-tuning on ISIC 2017, it obtained a Dice score of 91.69% ± 1.36% and an HD95 of *16.67 ± 2.73* pixels. On Kvasir-SEG, the model achieved a Dice score of 91.63% ± 0.57% and an HD95 of *25.87 ± 1.97* pixels. In a single-run component ablation, the complete model improved the Dice score by 0.96 percentage points and reduced HD95 by 1.16 pixels relative to the internal baseline. These results indicate that frequency-selective feature processing can help preserve weak or interrupted lesion boundaries.

## Introduction

Reliable lesion contours are central to dermoscopic assessment because border irregularity, lesion extent, and margin definition depend on accurate delineation [1]. However, in real dermoscopic images, the lesion boundary is generally the least stable part of the scene. Uneven illumination, color inhomogeneity, and weak local contrast can blur the contour, and hair can obscure portions of the contour [2], rendering precise segmentation difficult.

Earlier dermoscopic computer-aided diagnosis pipelines examined the selection and fusion of color, texture, shape, and deep features for lesion analysis [3, 4]. Subsequent segmentation networks have increasingly replaced manually selected features with end-to-end learned representations. Vision transformers have broadened the design space for medical-image segmentation by strengthening long-range context aggregation. Architectures such as TransUNet [5] and Swin-Unet [6, 7] combine long-range context modeling with hierarchical representations, which often improves lesion localization and region completeness [8]. However, spectral analyses indicate that self-attention tends to emphasize lower-frequency content [9]. In dermoscopic imagery, this bias can stabilize the global lesion region while leaving uncertain, weakly contrasting, or partially occluded boundaries underrepresented.

Boundary-aware methods introduce contour supervision or dedicated edge-processing branches to improve local delineation, whereas topology-aware approaches impose additional constraints on structural continuity [10, 11]. Recent skin lesion segmentation methods have addressed this issue from different perspectives. Att-Next combines modified ConvNeXt and attention blocks with a persistent homology-based topological loss [12]. This loss complements binary cross-entropy supervision by accounting for connected components and holes in the predicted lesion. Att-Next therefore addresses structural consistency primarily through its training objective rather than through frequency-specific processing of skip features.

Boundary delineation remains challenging because lesion appearance is not uniformly distributed across the spectrum. Coarse shapes and region-level semantics are largely concentrated in low-frequency components, whereas edge transitions and fine structural details are expressed more strongly in high-frequency responses [13, 14]. High-frequency components may also contain responses caused by hair, reflections, and image noise. Most segmentation networks learn these cues in the spatial domain, where useful boundary evidence and nuisance details are entangled. The discrete wavelet transform (DWT) separates a signal into an approximation band and several directional detail bands, and wavelet representations have been used for downsampling, restoration, denoising, and feature enhancement [15–17]. However, their effectiveness depends not only on the decomposition itself but also on how the resulting frequency bands are filtered and recombined. Without an additional filtering mechanism, skip connections may pass nuisance structures together with genuinely informative boundary details. This ambiguity motivates the use of low-frequency semantic information to guide the selection of high-frequency information before decoder fusion.

State-space models (SSMs) represented by Mamba [18] have recently emerged as efficient alternatives to attention for long-range sequence modeling. Visual variants such as U-Mamba [19] and VM-UNet [20] use selective scanning to maintain a large effective receptive field while controlling computational overhead. SM-UNet further combines axial-shift operations with a spatial SSM to fuse local and global features for skin lesion segmentation [21]. Existing visual state-space segmentation models generally apply state-space blocks within the main spatial feature hierarchy or use multiple scanning directions to cover a feature map [19, 20, 22]. However, frequency-specific refinement of skip features before decoder fusion has received less attention.

Thus, we developed Swin-WaveNet, a segmentation framework that considers boundary preservation as a frequency-guided restoration problem before decoding. The main contributions of this study are as follows.

1. We formulated skip-path processing as a pre-decoding boundary-restoration problem. Low-frequency semantic context was separated from high-frequency details, allowing the two components to be processed according to their different roles before reconstruction.
2. We designed a high-frequency selective state-space module (HF-SSM) that applies selective scanning to high-frequency subbands, enabling long-range interaction among detail responses without applying state-space modeling to the full feature map.
3. We proposed a wavelet-based dynamic fusion module (Wavelet-DFM) that uses semantic guidance, together with a high-frequency attention (HFA) decoder, to suppress artifact-related responses and preserve local boundary sharpness during upsampling.

## Methods

### Overall architecture

**Fig. 1:**
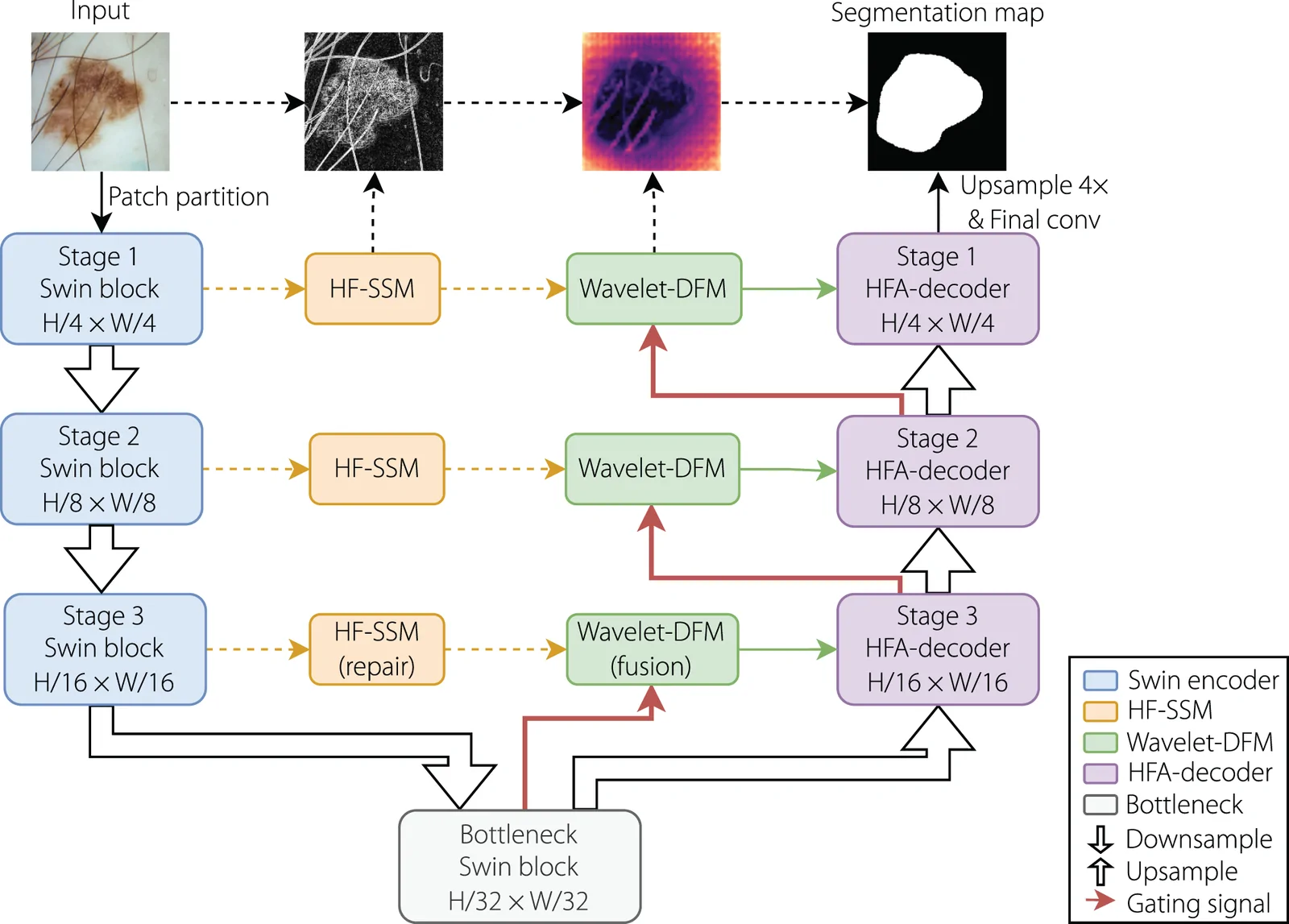
Overall architecture of Swin-WaveNet. Multiscale features from the Swin transformer encoder are decomposed using discrete wavelet transform in each skip branch. High-frequency selective state-space module refines the high-frequency subbands, wavelet-based dynamic fusion module suppresses artifact-related responses under semantic guidance, and the high-frequency attention decoder restores boundary detail before final prediction

An overview of Swin-WaveNet is shown in Fig. 1. The network follows a hierarchical U-shaped design but changes the manner in which skip features are handled before they reach the decoder. A Swin transformer encoder [7], initialized with ImageNet-pretrained weights, extracts multiscale semantic representations. Rather than forwarding the encoder features directly across skip connections, each skip branch is decomposed using the DWT and refined before reconstruction. The goal was to repair and filter boundary-related details before they are reintroduced during decoding.

In the proposed design, the HF-SSM reconnects broken edge responses in the high-frequency bands, wavelet-DFM filters out artifact-dominated activations with deeper semantic guidance, and the HFA decoder reinforces the local continuity during upsampling. This sequence allows contour recovery to take place before the final spatial reconstruction stage.

To match the unified *384 × 384* input resolution used throughout this study, the Swin encoder in the proposed framework and all ablation variants used a window size of 12. With a patch size of 4, the hierarchical feature resolutions became 96, 48, 24, and 12, all of which are exactly divisible by 12. This avoids irregular window partitioning under the *384 × 384* setting and maintains the internal consistency of the multistage encoder configuration.

### HF-SSM: selective repair in high-frequency bands

**Fig. 2:**
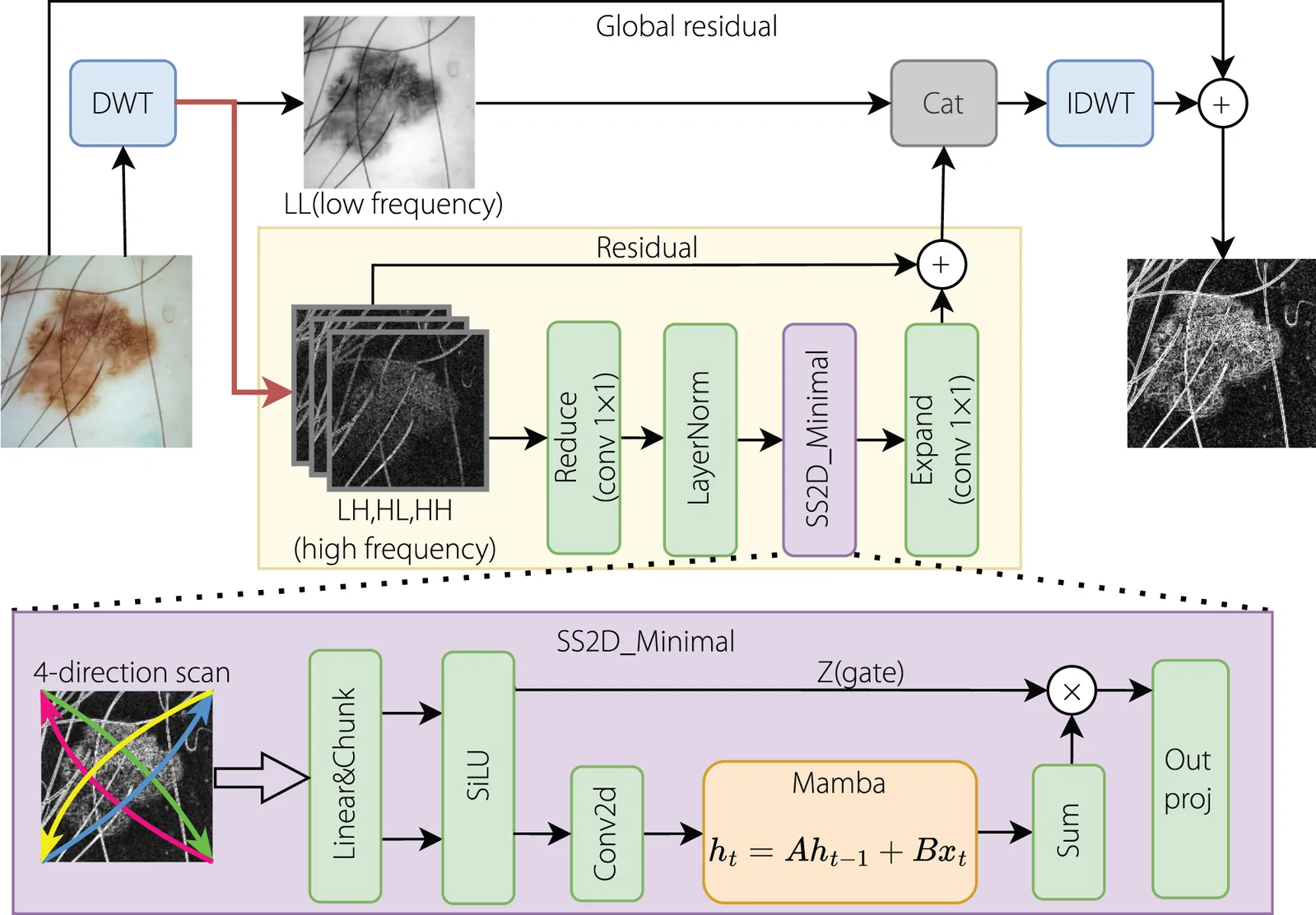
High-frequency selective state-space module. Discrete wavelet transform decomposes the input into one low-frequency band and three high-frequency subbands. Selective scanning was applied only to the high-frequency stream, whereas the low-frequency branch was bypassed and later recombined by inverse discrete wavelet transform

After several encoder stages, thin lesion borders may become fragmented because downsampling weakens the sparse edge responses. Mamba-based SSMs can propagate information over long ranges [18]. However, applying the same operation to an undifferentiated spatial feature map does not distinguish the coarse semantic structures from the boundary-related details. Therefore, we applied selective state-space modeling only to the high-frequency subbands, as illustrated in Fig. 2.

Using an input feature map *X*, we first applied a parameter-free Haar wavelet transform to obtain four subbands: $X_{LL}$, $X_{LH}$, $X_{HL}$, and $X_{HH}$. The approximation band $X_{LL}$ preserved coarse structural information, whereas $X_{LH}$, $X_{HL}$, and $X_{HH}$ retained the directional details that were more relevant to contour recovery. Therefore, we maintained the low-frequency branch constant and performed state-space modeling only on the concatenated high-frequency stream.

The three high-frequency subbands were projected onto a latent space and scanned using the two-dimensional selective scan (SS2D) [23], which unfolded a two-dimensional feature map into four one-dimensional sequences along different directions. This enabled the Mamba core to propagate information along interrupted contours and reconnect weakened responses. A learnable scale *Γ* controlled the magnitude of the high-frequency correction. The refined representation was then recombined with the preserved approximation band using the inverse discrete wavelet transform (IDWT):

1 $$X_{\mathrm{rec}}=\mathrm{IDWT}\left(X_{LL},X_{\mathrm{high}}+\Gamma \odot\mathrm{SS2D}(X_{\mathrm{high}})\right)$$

The reconstructed branch was then combined with the original spatial feature through the outer shortcut shown in Fig. 2.

2 $$X_{\mathrm{out}}=X+X_{\mathrm{rec}}$$

### Wavelet-DFM: semantic-guided noise gating

**Fig. 3:**
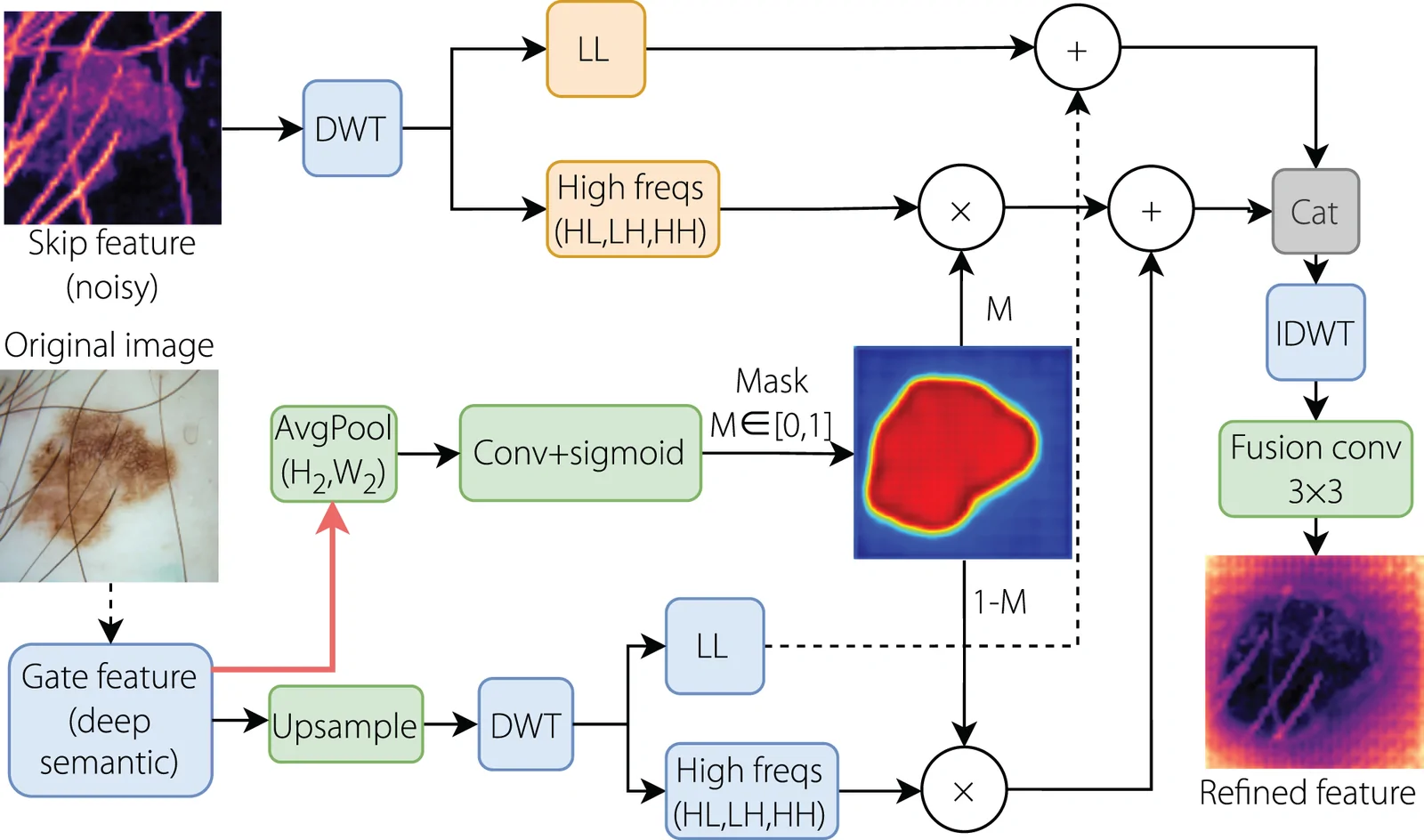
Wavelet-based dynamic fusion module. A shallow skip feature and a deeper semantic feature were decomposed into wavelet subbands. A confidence mask predicted from the semantic branch gated shallow high-frequency detail and replaced unreliable responses before inverse discrete wavelet transform reconstruction and skip fusion

A practical difficulty in dermoscopic segmentation was that the high-frequency bands contained both lesion edges and artifact-induced structures. For example, hair may generate sufficiently strong gradients to pass through a skip connection if no filtering is applied [2]. To reduce this effect, Wavelet-DFM injected semantic guidance into the frequency fusion process, as shown in Fig. 3.

*S* denotes a shallow skip feature and *G* denotes a deeper semantic feature. Both were decomposed into wavelet subbands. A confidence mask *M ∈ [0,1]* was predicted from *G* to identify the locations supported by deeper semantics:

3 $$M = \sigma\bigl(\mathrm{Conv}_{1\times1}(\mathrm{ReLU}(\mathrm{Conv}_{3\times3}(\mathrm{AvgPool}(G))))\bigr)$$

where *σ* denotes a sigmoid function. A mask was used to gate the shallow high-frequency components.

4 $$F_{\mathrm{high}} = S_{\mathrm{high}} \odot M + G_{\mathrm{high}} \odot (1-M)$$

When the semantic support was weak, *M* approached zero and noisy shallow gradients were attenuated. The complementary term injected the boundary-consistent details from $G_{\mathrm{high}}$. Low-frequency components were fused with a learned soft gate; therefore, skip fusion was driven by structural relevance rather than the raw gradient magnitude.

### HFA decoder: restoring local continuity during upsampling

**Fig. 4:**
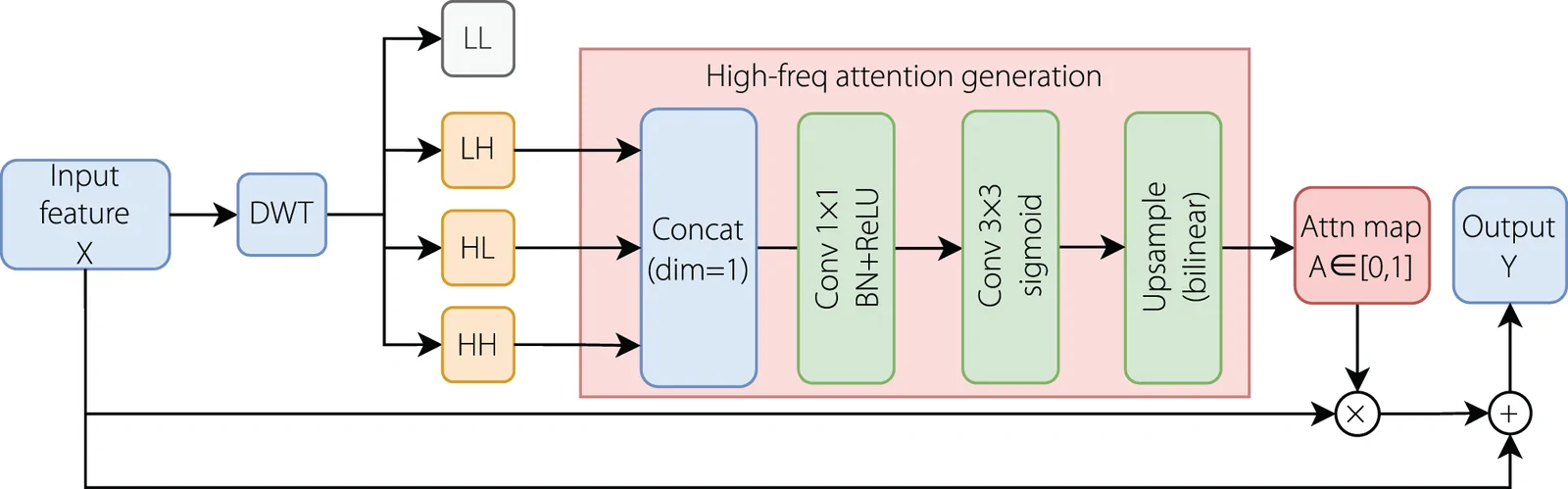
High-frequency attention decoder. The decoder feature was first upsampled by transposed convolution and concatenated with the corresponding skip feature. After convolutional fusion, a discrete wavelet transform-based attention branch extracted high-frequency responses, generated an attention map, and recalibrated the fused decoder feature

The patch-expanding decoders used in pure transformer architectures, including Swin-Unet [6], reconstructed the feature map through token expansion rather than overlap-aware convolution. In practice, this may leave the local continuity weakly constrained around the lesion boundary. Therefore, we adopted a decoder that combines transposed convolution with HFA, as shown in Fig. 4.

First, the linear upsampling was replaced by a transposed convolution (*2 × 2*, stride 2) [24]. The upsampled decoder feature was concatenated with the corresponding skip feature and then processed using pointwise and depthwise convolutions.

Second, to offset the smoothing introduced by the convolution, we added a HFA branch. High-frequency residuals were extracted from the fused decoder feature by DWT, and an edge attention map $M_{\mathrm{attn}}$ was produced as follows:

5 $$M_{\mathrm{attn}} = \sigma\bigl(\mathrm{Conv}(\mathrm{Concat}(X_{LH}, X_{HL}, X_{HH}))\bigr)$$

Before recalibration, the attention map $M_{\mathrm{attn}}$ was bilinearly upsampled to match the spatial resolution of *X*. The final decoder output was recalibrated as $Y = X \cdot (1 + M_{\mathrm{attn}})$, which selectively amplified the responses near the predicted boundary and facilitated maintaining sharper contour transitions.

### Datasets, training protocol, and statistical analysis

The model was evaluated using three public medical imaging datasets for complementary purposes. The International Skin Imaging Collaboration (ISIC) 2018 dataset [25] (2594 training images, 100 validation images, and 1000 test images) served as the primary benchmark for skin lesion segmentation. To assess the cross-dataset robustness, models trained on ISIC 2018 were evaluated directly on the 600-image test set of ISIC 2017 [26] without fine-tuning. Kvasir-SEG [27], which contained 1000 endoscopic images, was used as a modality-distinct benchmark to test whether the proposed design remains useful beyond dermoscopy. For Kvasir-SEG, each model was trained, validated, and tested on a fixed 7:1:2 split (700/100/200 images), without using ISIC images during training.

The comparisons included convolutional neural network-based models (UNet, AttnUNet, and SCANet), hybrid or transformer-based models (TransUNet, Swin-Unet, and HiFormer), and state-space- or topology-aware models (VM-UNet_V2, Att-Next [12], and SM-UNet [21]). All the baseline implementations were integrated into the same codebase and evaluated using the protocol described below.

All the models were implemented in PyTorch, and each training run was conducted on a single NVIDIA GeForce RTX 5090 GPU. All inputs were resized to *384 × 384* and normalized using the ImageNet mean and standard deviation. During the training, horizontal and vertical flips were applied with a probability of 0.5. Affine augmentation used a scale range of 0.9–1.1, a translation fraction of 0.1, and rotation within $\pm30^\circ$, with a probability of 0.5. One of the three distortions, elastic, grid, or optical, was applied with an overall probability of 0.3. Random brightness and contrast adjustments, Gaussian noise, and color jitter were applied with probabilities of 0.5, 0.2, and 0.3, respectively. The batch size was 32 for most models and four for TransUNet because of memory constraints. AttnUNet, SCANet, and Att-Next used a physical batch size of 16 with gradient accumulation over two steps, resulting in an effective batch size of 32.

The training used AdamW [28] with an initial learning rate of $5 \times 10^{-5}$ ($1 \times 10^{-5}$ for TransUNet) and cosine annealing [29] for 300 epochs, with early stopping after 50 epochs without validation improvement. The weight decay was 0.1, and the learning rate was linearly warmed for 20 epochs before the cosine decay to $1 \times 10^{-6}$. Swin Transformer backbones were initialized using ImageNet-pretrained weights [30]. The pretrained Swin-Tiny checkpoint was obtained at an input resolution of *224 × 224* with a window size of seven. During the experiments, both Swin-WaveNet and the standalone Swin-Unet used a runtime window size of 12 at *384 × 384*. The relative-position bias tables were interpolated when the pretrained weights were loaded, whereas the position indices and attention masks were regenerated for the new window configuration.

Except for Att-Next, the models were optimized using an equally weighted combination of binary cross-entropy and Dice losses. Att-Next used binary cross-entropy together with topology-aware loss, with a topology weight of 0.1. Att-Next and SM-UNet were trained with full precision, whereas mixed precision training was used for the remaining models.

The comparative results presented in Tables 1, 2 and 3 were obtained from three independent training runs with 3232, 3407, and 42 random seeds. In each run, the checkpoint that achieved the highest validation Dice score was used for evaluation. Tables 4 and 5 present the single-run results of the component and scanning-target analyses, respectively.

**Table 1:** Quantitative comparison using ISIC 2018

Model	Dice (%)	IoU (%)	Sensitivity (%)	Specificity (%)	HD95 (px)
UNet [33]	*86.99 ± 0.27*	*79.43 ± 0.55*	*89.80 ± 0.78*	$\boldsymbol{95.46 \pm 1.03}$	*37.82 ± 1.82*
AttnUNet [34]	*87.49 ± 0.60*	*79.82 ± 0.82*	*91.58 ± 0.94*	*94.51 ± 0.84*	*36.72 ± 2.47*
TransUNet [5]	*88.57 ± 0.27*	*81.56 ± 0.46*	*92.63 ± 0.32*	*94.36 ± 0.26*	*27.78 ± 0.33*
Swin-Unet [6]	*89.62 ± 0.41*	$\boldsymbol{82.74 \pm 0.45}$	*94.60 ± 0.69*	*93.89 ± 0.28*	*23.67 ± 0.22*
SCANet [35]	*86.45 ± 0.34*	*78.53 ± 0.34*	*91.78 ± 1.31*	*93.31 ± 1.23*	*36.31 ± 1.69*
HiFormer [36]	*89.34 ± 0.27*	*82.07 ± 0.46*	*95.11 ± 0.14*	*94.00 ± 0.46*	*24.40 ± 0.37*
VM-UNet_V2 [37]	*87.71 ± 0.19*	*80.42 ± 0.04*	*92.89 ± 0.37*	*93.30 ± 0.69*	*30.72 ± 1.17*
Att-Next [12]	*88.85 ± 0.36*	*81.79 ± 0.55*	*93.25 ± 0.29*	*93.87 ± 0.89*	*26.95 ± 0.54*
SM-UNet [21]	*85.22 ± 0.68*	*77.35 ± 1.48*	*90.48 ± 0.81*	*93.01 ± 0.94*	*37.29 ± 2.42*
**Swin-WaveNet (ours)**	$\boldsymbol{89.69 \pm 0.51}$	*82.46 ± 0.65*	$\boldsymbol{95.27 \pm 0.32}$	*93.43 ± 0.46*	$\boldsymbol{23.60 \pm 1.04}$

Values are reported as the mean *±* SD of the sample over three runs. Bold numerical values indicate the best mean result for each metric: higher Dice, IoU, sensitivity, and specificity, and lower HD95. HD95: 95th-percentile Hausdorff distance; IoU: Intersection over union

**Table 2:** Cross-dataset evaluation on ISIC 2017

Model	Dice (%)	IoU (%)	Sensitivity (%)	Specificity (%)	HD95 (px)
UNet	*83.25 ± 0.07*	*74.45 ± 0.20*	*79.04 ± 0.44*	$\boldsymbol{98.38 \pm 0.15}$	*36.90 ± 0.71*
AttnUNet	*86.15 ± 1.95*	*78.27 ± 2.81*	*82.90 ± 1.99*	*98.02 ± 0.14*	*32.00 ± 4.27*
TransUNet	*86.34 ± 1.33*	*78.18 ± 1.66*	*83.06 ± 0.85*	*97.85 ± 0.25*	*29.43 ± 0.89*
Swin-Unet	*87.64 ± 3.07*	*80.09 ± 4.09*	*82.93 ± 4.12*	*98.32 ± 0.14*	*21.48 ± 5.03*
SCANet	*86.47 ± 0.84*	*79.14 ± 1.26*	*83.28 ± 0.20*	*97.09 ± 0.24*	*30.88 ± 1.63*
HiFormer	*89.96 ± 3.39*	*83.57 ± 4.92*	*89.07 ± 3.55*	*98.27 ± 0.22*	*18.31 ± 5.57*
VM-UNet_V2	*86.50 ± 0.65*	*78.46 ± 0.80*	*83.21 ± 1.72*	*97.65 ± 0.10*	*27.07 ± 1.67*
Att-Next	*87.34 ± 0.99*	*79.43 ± 1.28*	*83.80 ± 1.12*	*97.61 ± 0.28*	*24.18 ± 1.22*
SM-UNet	*79.27 ± 0.65*	*69.91 ± 0.62*	*78.50 ± 2.44*	*96.70 ± 0.86*	*42.80 ± 1.28*
**Swin-WaveNet (ours)**	$\boldsymbol{91.96 \pm 1.36}$	$\boldsymbol{86.22 \pm 1.92}$	$\boldsymbol{89.84 \pm 2.20}$	*98.05 ± 0.52*	$\boldsymbol{16.67 \pm 2.73}$

All models were trained on ISIC 2018 and evaluated on ISIC 2017 without fine-tuning. Values are mean*±*standard deviation of the sample over three runs. Bold numerical values indicate the best mean result for each metric: higher Dice, IoU, sensitivity, and specificity, and lower HD95. HD95: 95th-percentile Hausdorff distance; IoU: Intersection over union

**Table 3:** Evaluation using Kvasir-SEG

Model	Dice (%)	IoU (%)	Sensitivity (%)	Specificity (%)	HD95 (px)
UNet	*82.25 ± 0.16*	*73.14 ± 0.11*	*84.90 ± 0.97*	*97.40 ± 0.30*	*65.84 ± 4.50*
TransUNet	*78.80 ± 0.22*	*69.89 ± 0.23*	*79.32 ± 0.50*	*97.53 ± 0.26*	*55.94 ± 1.64*
Swin-Unet	*91.23 ± 0.22*	*85.68 ± 0.33*	*90.75 ± 0.95*	$\boldsymbol{98.91 \pm 0.15}$	*26.13 ± 0.58*
**Swin-WaveNet (ours)**	$\boldsymbol{91.63 \pm 0.57}$	$\boldsymbol{86.31 \pm 0.76}$	$\boldsymbol{92.75 \pm 0.32}$	*98.56 ± 0.17*	$\boldsymbol{25.87 \pm 1.97}$

All models used the same 7:1:2 train/validation/test split. The values are the mean*±*standard deviation of the sample over three runs. Bold numerical values indicate the best mean result for each metric: higher Dice, IoU, sensitivity, and specificity, and lower HD95. HD95: 95th-percentile Hausdorff distance; IoU: Intersection over union

**Table 4:** Component ablation using ISIC 2018

ID	HF-SSM	Wavelet-DFM	HFA decoder	Dice (%)	IoU (%)	Sensitivity (%)	Specificity (%)	HD95 (px)
B	*×*	*×*	*×*	88.74	81.19	94.91	93.35	24.79
B1	*\checkmark*	*×*	*×*	89.22	81.91	95.12	93.84	24.13
B2	*×*	*\checkmark*	*×*	88.88	81.50	94.78	**93.90**	24.71
B12	*\checkmark*	*\checkmark*	*×*	89.27	81.99	**95.55**	92.52	24.30
B123	*\checkmark*	*\checkmark*	*\checkmark*	**89.70**	**82.65**	95.32	93.29	**23.63**

B denotes the internal ablation baseline and is not the standalone Swin-Unet comparator. Results are from a single run for each configuration. *\checkmark* and *×* indicate enabled and disabled components, respectively. Bold numerical values indicate the best result for each metric: higher Dice, IoU, sensitivity, and specificity, and lower HD95. HD95: 95th-percentile Hausdorff distance; IoU: Intersection over union

**Table 5:** Effect of scanning targets on ISIC 2018

Scanning target	Params (M)	GFLOPs	Dice (%)	IoU (%)	Sensitivity (%)	Specificity (%)	HD95 (px)
Low-frequency	**35.29**	**27.74**	88.23	80.54	95.26	92.21	26.45
Full feature map	**35.29**	27.91	89.31	82.15	95.10	93.07	24.50
High-frequency	37.90	28.19	**89.70**	**82.65**	**95.32**	**93.29**	**23.63**

Results are from a single run for each configuration. Params (M) denotes the number of parameters in millions. GFLOPs denotes giga floating-point operations, with one multiply-accumulate operation counted as one operation, consistent with the efficiency comparison. Bold numerical values indicate the best value in each column, including ties: lower parameter count, GFLOPs, and HD95, and higher Dice, IoU, sensitivity, and specificity. HD95: 95th-percentile Hausdorff distance; IoU: Intersection over union

All comparison models were evaluated under a unified protocol within each benchmark, using the same dataset partition, *384 × 384* input resolution, metric implementation, and a fixed probability threshold of 0.5. The threshold was not adjusted for the cross-dataset evaluation on ISIC 2017, and no connected-component filtering, morphological refinement, or other postprocessing was applied. The Dice score, intersection over union (IoU), sensitivity, specificity, and the 95th-percentile Hausdorff distance (HD95) were calculated for each test image. HD95 was computed as the symmetric 95th-percentile distance between the surfaces of the predicted and reference masks and reported in pixels. When both masks were empty, HD95 was set to zero; when only one mask was empty, an image diagonal was used.

For the statistical analysis, each metric was averaged over the test set within each run. Comparative results were reported as the mean and standard deviation of the sample across the three seeds. Two-sided 95%CIswere calculated across runs using the Student’s *t* distribution with two degrees of freedom. The test images were not treated as independent experimental replicates.

## Results

### Performance on ISIC 2018

Quantitative comparisons of nine representative methods are presented in Table 1. Dice score measures region overlaps; however, it is relatively insensitive to small local contour errors. Therefore, we considered the HD95 as an informative boundary metric [31, 32]. The main comparison reported the mean and standard deviation of the sample over three runs with seeds 3232, 3407, and 42.

On ISIC 2018, Swin-WaveNet obtained a mean Dice score of 89.69% and a mean HD95 of 23.60 pixels. These were numerically the highest Dice score and the lowest HD95 presented in Table 1, although the differences from Swin-Unet were small. Swin-WaveNet achieved the highest mean sensitivity of 95.27%. Swin-Unet achieved a slightly higher IoU of 82.74%, whereas UNet achieved the highest specificity of 95.46%. The 95%CIs across the three runs for Swin-WaveNet were 88.42%–90.96% for Dice score and 21.02–26.18 pixels for HD95.

**Fig. 5:**
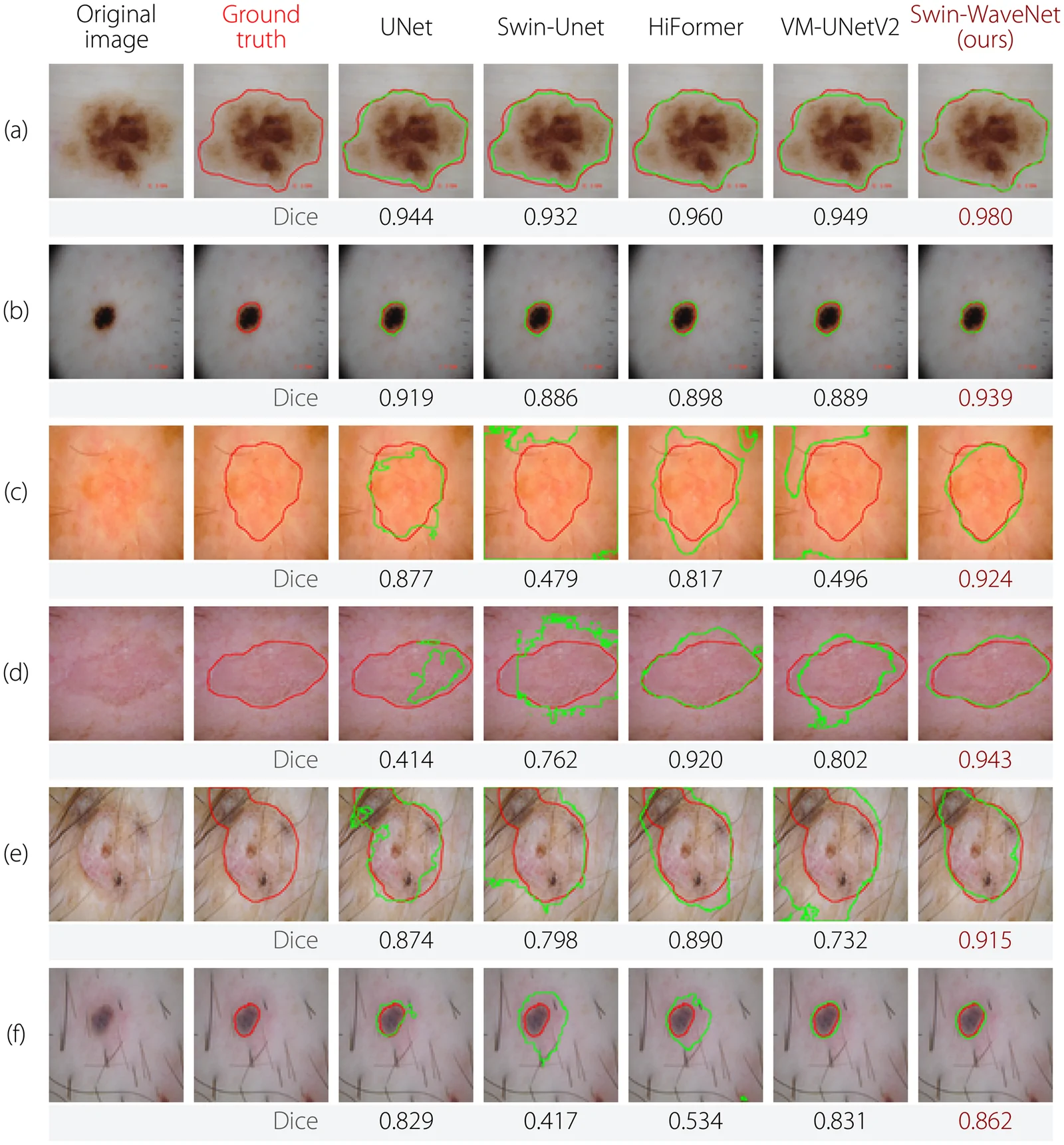
Qualitative comparison using ISIC 2018. Red contours represent ground truth, and green contours represent model predictions. Cases (**a**), (**b**) are typical lesions, (**c**), (**d**) highlight weak or blurred margins, and (**e**), (**f**) show strong hair interference

In the examples shown in Fig. 5, Swin-WaveNet produced more complete contours in several cases with weak borders or hair occlusion and reduced some peripheral false-positive responses.

### Cross-dataset evaluation on ISIC 2017

To test the robustness of the cross-dataset, the models trained on ISIC 2018 were evaluated directly on ISIC 2017 without fine-tuning. The same fixed threshold of 0.5 was used to binarize the predictions, and no threshold adjustment or postprocessing was applied during cross-dataset testing. Swin-WaveNet achieved the highest mean Dice score and IoU and the lowest mean HD95, reaching a Dice score of 91.69% ± 1.36% and HD95 of *16.67 ± 2.73* pixels (Table 2). The 95%CIs across the three runs were 88.58%–95.34% for Dice score and 9.89–23.45 pixels for HD95.

One possible reason is that frequency decomposition partially separates the appearance variation from the structural details. Previous studies on frequency-based domain adaptation suggested that changes in illumination and image style are often more strongly concentrated in lower-frequency components, whereas some structural information remains relatively stable across domains [38, 39]. This frequency-based separation may contribute to the robustness of the cross-dataset evaluation.

### Evaluation on the modality-distinct Kvasir-SEG benchmark

Kvasir-SEG introduced a substantial difference in modality because it consisted of endoscopic rather than dermoscopic images. Therefore, we used it to test whether the proposed boundary restoration design remained effective outside the skin-lesion segmentation. For this benchmark, all the models were trained and evaluated using the same within-dataset split described above.

Under this protocol, Swin-WaveNet achieved a Dice score of 91.63% ± 0.57% and an HD95 of *25.87 ± 1.97* pixels, whereas Swin-Unet achieved a Dice score of 91.23% ± 0.22% and an HD95 of *26.13 ± 0.58* pixels (Table 3). Swin-WaveNet also exhibited a mean sensitivity of 92.75%, whereas Swin-Unet achieved the highest specificity of 98.91%. These results confirm the effectiveness of frequency-guided boundary restoration for endoscopic polyp segmentation. The 95%CIs across the three runs for Swin-WaveNet were 90.21%–93.05% for Dice score and 20.98–30.76 pixels for HD95.

### Ablation studies

#### Contribution of the main components

Table 4 presents the results of the internal ablation study. Here, B denotes the plain model within the unified *384 × 384* framework, which retained the same Swin encoder setting (window size 12) and the same convolutional decoder as the proposed architecture, whereas HF-SSM, Wavelet-DFM, and HFA were all disabled. Therefore, B should be interpreted as the internal ablation reference rather than as the standalone Swin-Unet baseline presented in Table 1.

In this single-run comparison, adding the HF-SSM changed the Dice score from 88.74% to 89.22% and HD95 from 24.79 to 24.13 pixels. Adding Wavelet-DFM increased the specificity from 93.35% to 93.90%. When the HFA decoder was added to both modules, the Dice score changed from 89.27% to 89.70%, and HD95 from 24.30 to 23.63 pixels. Relative to model B, the complete model increased the Dice score by 0.96 percentage points and reduced HD95 by 1.16 pixels.

#### Choice of scanning target

To test the relevance of scanning target, we compared three HF-SSM variants (Table 5). Scanning only the low-frequency branch was the least effective method for boundary recovery, yielding an HD95 of 26.45 pixels. Scanning the full feature map improved the performance, reducing HD95 to 24.50 pixels. Restricting scanning to high-frequency subbands produced the best result, with a Dice score of 89.70%, an IoU of 82.65%, and an HD95 of 23.63 pixels.

Compared with full-feature scanning, the high-frequency configuration increased the Dice score by 0.39 percentage points and reduced HD95 by 0.87 pixels. Concatenating the three high-frequency subbands increased the input dimension from *C* to *3C*, resulting in 2.61 million more parameters and 0.28 additional giga floating-point operations (GFLOPs). Although this capacity difference prevented the complete isolation of the scanning target, the high-frequency variant achieved the best Dice score and HD95 among the evaluated configurations.

## Discussion

The experimental results suggest that the performance of Swin-WaveNet is related not only to the model capacity, but also to the manner in which boundary-related features are processed. On ISIC 2018 and Kvasir-SEG, Swin-WaveNet achieved a performance comparable to that of Swin-Unet, while exhibiting a higher mean Dice score and lower HD95 in the cross-dataset evaluation using ISIC 2017. Component and scanning-target ablations were also associated with a lower HD95 when the high-frequency subbands were refined. These results motivate the frequency-guided skip feature refinement prior to decoder reconstruction.

Recent methods address boundary integrity from different perspectives. Att-Next introduces topological awareness through its training objective [12], whereas SM-UNet combines local and global information by using axial shifting and spatial state modeling [21]. Swin-WaveNet differs from these methods in that it decomposes skip features into frequency components and applies selective state-space modeling to boundary-dominant high-frequency subbands. Its main contributions lie in coordinating wavelet decomposition, selective scanning, semantic-guided fusion, and decoder refinement around a predecoding boundary restoration process.

### Frequency-domain separation and boundary recovery

In the spatial domain, true lesion borders and acquisition artifacts are both expressed as local gradients, making it difficult to disentangle them using purely local filters, particularly when the contour is faint. Wavelet decomposition narrows this problem by partitioning the feature map into an approximation band and several directional detail bands [17]. High-frequency components contain both useful and nuisance edges; however, the search space for boundary refinement has become more focused.

The ablation results supported this interpretation. Wavelet-DFM alone increased the specificity from 93.35% to 93.90%. When all three proposed modules were used, HD95 decreased from 24.79 to 23.63 pixels relative to the internal baseline. The scanning-target experiment also demonstrated that processing the high-frequency subbands produced a lower HD95 than scanning either the low-frequency component or the full feature map, although the high-frequency variant contained more parameters.

### Continuity modeling with selective state-space scanning

Self-attention connects positions using pairwise similarities [40]. This is useful for semantic grouping; however, it does not explicitly encourage a contour to remain continuous from one location to another. The HF-SSM introduces a different inductive bias, in which information is recursively propagated along the scan directions. In the SS2D setting [23], this allows the boundary evidence to pass across short interruptions and reinforces nearby weak segments.

6 $$h_t = A h_{t-1} + B x_t$$

When the HF-SSM was added to the internal baseline, the Dice score increased by 0.48 percentage points and HD95 decreased by 0.66 pixels. High-frequency scanning also reduced HD95 by 0.87 pixels relative to full-feature scanning. These single-run results provide limited empirical support for the intended role of selective state-space propagation in connecting weak or interrupted boundary responses.

### Computational cost and wavelet overhead

**Fig. 6:**
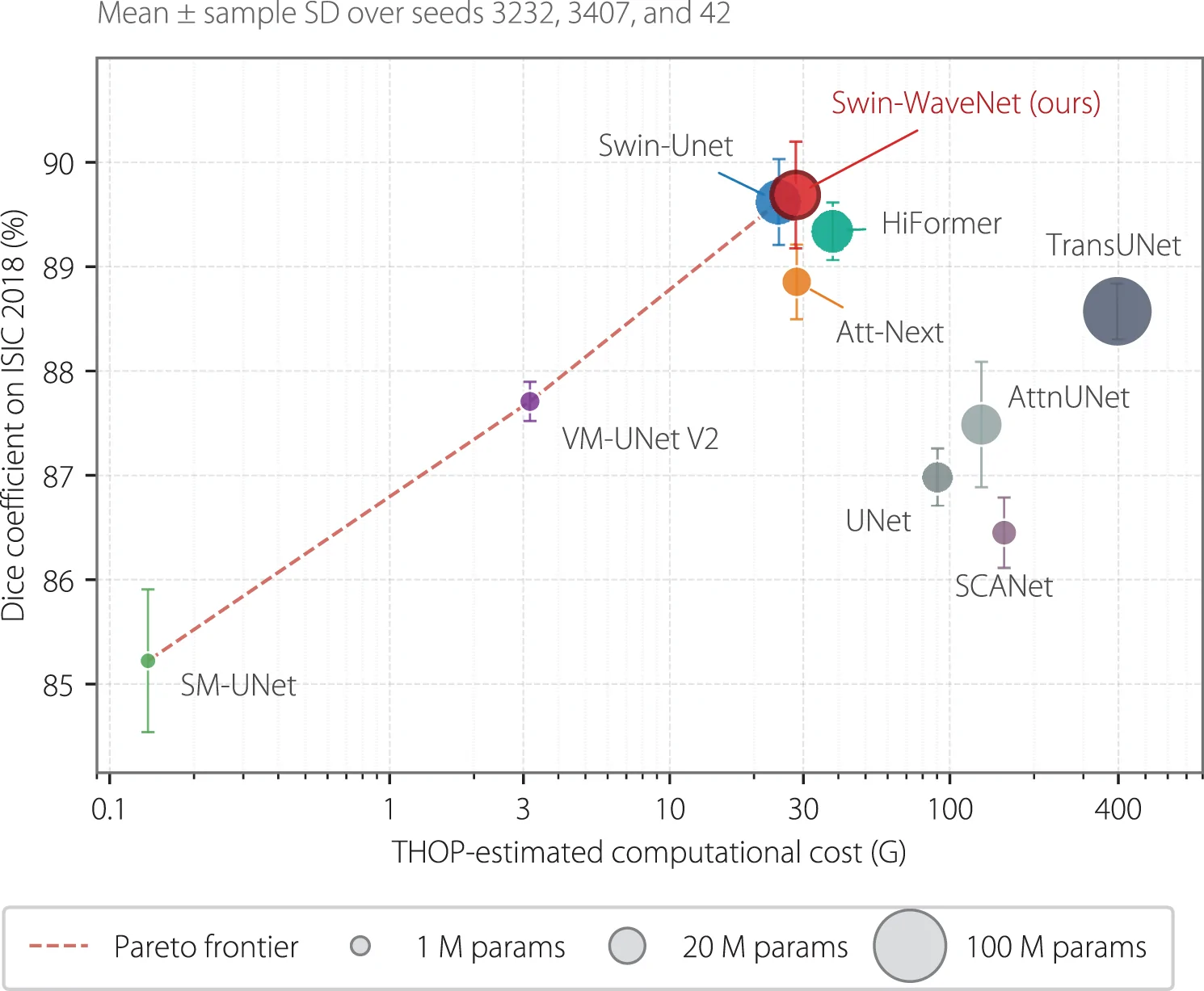
Dice-giga floating-point operations comparison on ISIC 2018. Points show the mean Dice score over three runs, and vertical error bars indicate *±* one standard deviation. Bubble size represents the number of parameters, with a minimum display size applied to small models. The dashed line marks the Pareto frontier for higher Dice and lower computational cost. GFLOPs denotes giga floating-point operations, with one multiply-accumulate operation counted as one operation

The computational efficiency is important when segmentation models are used for broader analytical pipelines. Figure 6 compares the models on the Dice-GFLOPs plane. Swin-WaveNet consisted of 37.90 million parameters and required 28.19 GFLOPs for a *384 × 384* input under the one-multiply-accumulate operation-one-operation convention. It is not the lowest-cost model in the comparison; its computation is higher than that of Swin-Unet (24.55 GFLOPs), closely similar to that of Att-Next (28.40 GFLOPs), and substantially lower than that of TransUNet (396.52 GFLOPs).

For comparison, TransUNet required 98.53 million parameters, whereas HiFormer required 36.40 million parameters and 38.22 GFLOPs. Swin-Unet provided a similar mean Dice score with a lower computational cost, although its parameter count was higher at 41.35 million. Att-Next required 28.40 GFLOPs and obtained a mean Dice score of 88.85%.

In Swin-WaveNet, state-space processing is confined to frequency components that are more likely to contain boundary evidence. This design focuses on the allocation of modeling capacity rather than a reduction in the total computation.

We also measured the costs specifically introduced by the Haar DWT and IDWT operations. Latency was measured using 32-bit floating-point precision with a batch size of one. Ten repeated trials were performed after 50 warm-up iterations, with each trial averaging 100 forward passes timed using Compute Unified Device Architecture events. For single-image inference at *384 × 384* on an NVIDIA GeForce RTX 5090, the complete model required *12.828 ± 0.123* ms. A shape-matched control that retained the remaining HF-SSM computation but omitted the explicit Haar transforms required *12.071 ± 0.109* ms. The DWT/IDWT path therefore added approximately 0.757 ms, corresponding to 5.90% of the complete inference time.

### Limitations

**Fig. 7:**
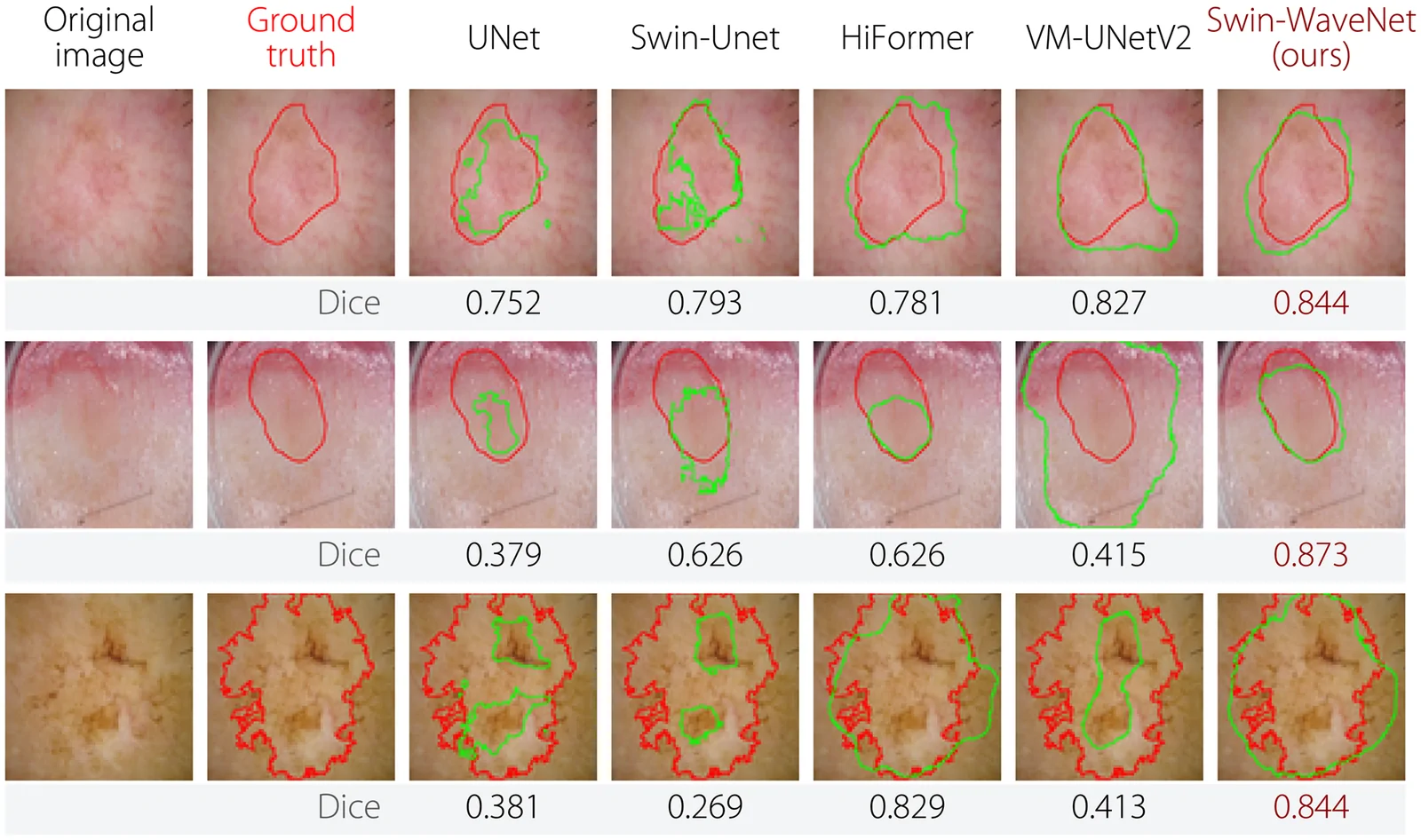
Challenging examples involving diffuse or low-contrast lesion boundaries. Red contours represent ground truth, and green contours represent model predictions. Dice scores are shown for the individual predictions

Diffuse lesions with gradual pigmentation changes remain challenging to segment because their lesion-to-background transitions contain limited high-frequency evidence. Under these conditions, HF-SSM and the HFA decoder receive less structural information for boundary refinement. Representative examples of diffuse or low-contrast lesion boundaries are shown in Fig. 7. This limitation suggests that contrast enhancement, uncertainty-aware refinement, or additional structural priors may be useful when the available high-frequency details are insufficient.

The evaluation was limited to three public datasets and three training runs. Further validation on multicenter data acquired using different imaging devices can provide a more complete assessment of generalizability. In addition, the proposed model contains 37.90 million parameters, and a lighter encoder may be preferable for deployment in resource-constrained devices.

## Conclusions

Swin-WaveNet reframes skin lesion segmentation as a boundary restoration problem that can be addressed prior to spatial reconstruction. By decomposing the encoder features using wavelets, the model separates coarse lesion structure from boundary-dominant details and applies selective state-space scanning only to the latter [16–18, 23]. Therefore, skip features are not merely passed forward; they are repaired, filtered, and selectively emphasized.

Swin-WaveNet achieved a mean Dice score of 89.69% with an HD95 of 23.60 pixels on ISIC 2018. In the cross-dataset evaluation using ISIC 2017 without fine-tuning, a Dice score of 91.96% and an HD95 of 16.67 pixels were obtained. Using Kvasir-SEG, it obtained a Dice score of 91.63% and an HD95 of 25.87 pixels. The component ablation study demonstrated that the complete model increased the Dice score by 0.96 percentage points and reduced the HD95 by 1.16 pixels relative to the internal baseline. Among the three scanning variants, the high-frequency configuration produced the highest Dice score and the lowest HD95, even though it used more parameters than the low-frequency and full-feature variants.

However, this method is less effective when the lesion-to-background transition contains insufficient high-frequency boundary evidence. Future study will examine additional structural priors, broader multicenter validation, and lighter model configurations for resource-constrained deployment. Overall, the results support frequency-guided boundary refinement as a useful complement to transformer-based medical-image segmentation.

## Data Availability

The datasets analyzed in this study are publicly available from the ISIC Challenge repository for ISIC 2017 lesion segmentation and ISIC 2018 lesion boundary segmentation, and the Simula Kvasir-SEG repository (https://challenge.isic-archive.com/landing/2017/42/,https://challenge.isic-archive.com/landing/2018/45/,https://datasets.simula.no/kvasir-seg/). The implementation used in this study is publicly available in the Swin-WaveNet GitHub repository (https://github.com/run619814/Swin-WaveNet).

## Abbreviations

DWT: Discrete wavelet transform
GFLOP: Giga floating-point operation
HD95: 95th-percentile Hausdorff distance
HFA: High-frequency attention
HF-SSM: High-frequency selective state-space module
IDWT: Inverse discrete wavelet transform
IoU: Intersection over union
ISIC: International Skin Imaging Collaboration
SS2D: Two-dimensional selective scan
SSM: State-space model
Wavelet-DFM: Wavelet-based dynamic fusion module
